# Gated recurrent unit model for forecasting greenhouse gas concentrations with uncertainty quantification

**DOI:** 10.3389/frai.2026.1782333

**Published:** 2026-06-22

**Authors:** Erica Hargety Kimei, Devotha Godfrey Nyambo, Neema Mduma, Shubi Felix Kaijage

**Affiliations:** 1 School of Computation and Communication Science and Engineering, The Nelson Mandela African, Institution of Science and Technology, Arusha, Tanzania; 2 Informatics and Technical Education, National Institute of Transport, Dar-Es-Salam, Tanzania

**Keywords:** forecasting, GHG, greenhouse gas concentration, GRU, IoT, remote sensing, uncertainty quantification

## Abstract

Reliable and accurate farm-level forecasting of greenhouse gas concentrations from dairy cattle is important to the formulation of climate change mitigation strategies and policy planning in livestock systems. This study proposed an uncertainty-aware deep learning model that integrated data from multiple sources, including remote sensing and ground-based sensors, to forecast hourly concentrations of nitrous oxide, methane, and carbon dioxide in a controlled zero-grazing dairy system. The forecasting was implemented by formulating a causal multivariate time series using a 24-h lookback window with direct one-step-ahead prediction. A two-stage model evaluation protocol, model hyperparameter tuning via rolling cross-validation, and holdout Test Evaluation were implemented during model development. The model was evaluated using the coefficient of determination, root-mean-square error, and mean squared error. To evaluate model reliability, the study employed a dual-output gated recurrent unit to estimate the conditional mean and heteroscedastic variance, and Monte Carlo dropout and Gaussian Negative Log-Likelihood to quantify epistemic and aleatoric uncertainty. Results indicate stable generalisation across temporal folds and strong probabilistic calibration, with empirical 95% coverage ranging from 93.6 to 94.8% on the holdout test set. Feature selection indicates that rainfall, normalised difference vegetation index, humidity, temperature, trend, and season influence the prediction of concentration. The study shows that incorporating exogenous variables improves model performance. The proposed framework demonstrates proof of concept for controlled zero-grazing systems, with potential for broader application following multi-site validation.

## 1 Introduction

Greenhouse gases (GHG), such as nitrous oxide (N₂O), carbon dioxide (CO₂), and methane (CH₄), are the primary sources of climate change, resulting in increased global warming, land degradation, air and water pollution, and declines in biodiversity (Hastomo et al., 2022; Munier et al., 2020). These gases can be generated from natural or anthropogenic activities (Cusworth et al., 2020; Gautam et al., 2021). Statistically, ruminant livestock contributes 14.5% of global greenhouse emissions through manure management and enteric fermentation (Bekele et al., 2022; Chand, 2022; Ntinyari and Gweyi-Onyango, 2021). Dairy cattle contribute approximately 4% of total global GHG emissions. In Sub-Saharan Africa, agriculture accounts for 5 to 15% of the economy and 66% emissions (Zhao et al., 2025). In Tanzania, dairy cattle generate approximately 28.8 million tonnes of CO₂ equivalent, with manure management contributing 8.2% and enteric methane accounting for 91.4% of the total emissions. This highlights the need to develop mitigation strategies to reduce emissions, thereby supporting both climate goals and food security (FAO and New Zealand Agricultural Greenhouse Gas Research Centre, 2019). Despite dairy’s contribution to GHG emissions, climate change also affects the dairy sector through rising temperatures, declines in feed quantity and quality, water scarcity, and new pests and diseases that threaten it (Chelang’a et al., 2025). These dual changes have compelled Sub-Saharan African nations to develop climate change policies to mitigate the impact of GHG emissions, and these policies have been incorporated into national frameworks that align with the Paris Agreement’s long-term objectives (Mwaura et al., 2024). Tanzania has set ambitious targets to reduce its economy-wide greenhouse gas emissions by 30–35% by 2030, which aligns with the Paris Agreement’s long-term objectives (URT, 2021). Studies on the dairy sector suggest that effective GHG mitigation strategies should be implemented at the farm level, where traditional farming knowledge can be integrated to improve mitigation (Chatrabhuj et al., 2025). This includes improved manure and herd management, feed improvement strategies, and feed conservation (Dawite et al., 2025; Kihoro et al., 2021). Various tools, such as the Global Livestock Environmental Assessment (GLEAM), the Intergovernmental Panel on Climate Change (IPCC), and Life Cycle Assessment (LCA), have been used to quantify greenhouse gas emissions (IPCC, 2019; Takahashi et al., 2020). East Africa relies on the default IPCC Tier 1 and 2 methodologies for data collection and estimation of GHG emissions per county. The farming systems in these countries are unique and diverse, with variations in breeds, climates, and feeding practices. The default assumptions and values are often invalid; furthermore, estimates of GHG emissions are overestimated and highly uncertain (Merbold et al., 2019). Life Cycle Assessment and GLEAM utilised assumptions regarding feed imports and manure handling, which limit comparability and introduce data gaps in smallholder systems, thereby reducing precision in Sub-Saharan Africa, as reported by GLEAM (Baker and Axon, 2025; Mazzetto et al., 2022). There is a need for localised measurements to detect, accurately, quantify, monitor, and analyse GHG concentrations at the farm level, thereby enabling enhanced evidence-based emission mitigation strategies. The information obtained will help to meet the goal of reporting credible national data under the Paris Climate Agreement and identifying tailored mitigation plans (Chauhan, 2020; Hasan et al., 2025; Leitner et al., 2020).

Thus, the emergence of technology such as artificial intelligence (AI), machine learning (ML), deep learning (DL), remote sensing, and the Internet of Things can be used to monitor, measure, and estimate GHG emissions at the farm level (Cusworth et al., 2020). These technologies can capture, identify, and understand the primary contributors of GHG emissions at the farm level (Ghassemi Nejad et al., 2024). These technologies enhance temporal and spatial coverage and enable modelling of nonlinear relationships. They also support scenario simulations that assist policymakers in designing tailored mitigation strategies and climate adaptation pathways (Hasan et al., 2025). Forecasting techniques analyse historical data to predict future trends (Arndt et al., 2022). These methods transform sequential time-series data into supervised learning problems by using historical observations to predict future values (Ene Yalçın, 2024; Hamdan et al., 2020). Incorporation of uncertainty quantification (UQ) into the forecasting results is vital for the accuracy and reliability of the model results (Cartolano et al., 2024; Singh et al., 2024; Wang et al., 2025).

Uncertainty quantification techniques like Bayesian deep learning and Monte Carlo (MC) dropout ensure the reliability of model results by providing confidence intervals and risk bounds (Singh et al., 2024). These techniques improve model transparency and reliability for policymakers and stakeholders. This study proposes integrating deep learning with the Internet of Things, enhanced by uncertainty quantification, to provide a more interpretable, robust, and reliable framework for environmental governance.

Different machine learning, deep learning, and statistical models, such as Exponential Smoothing and Auto-Regressive Integrated Moving Average (ARIMA), Long Short-Term Memory (LSTM), Support Vector Machine (SVM), Random Forest (RF), Bidirectional Long Short-Term Memory (BiLSTM), and Gated Recurrent Unit (GRU), can be used to forecast greenhouse gas emissions (Zhang, 2023). Forecasting provides policymakers and stakeholders with critical insights and support in decision-making (Ene Yalçın, 2024; Hamdan et al., 2020).

Emami Javanmard and Ghaderi (2022) developed a hybrid model combining artificial neural networks (ANN), LSTM, K-nearest neighbours (KNN), SVR, RF, autoregressive (AR), ARIMA, Seasonal Autoregressive Integrated Moving Average (SARIMA), and Seasonal Autoregressive Integrated Moving Average with eXogenous regressors (SARIMAX), along with optimisation models such as Particle Swarm Optimisation (PSO) and Grey Wolf Optimiser (GWO) to forecast CO₂, N₂O, CH₄, and fluorinated gases. The study utilised a dataset from the Iranian energy market spanning from 1990 to 2018 and employed stepwise regression to examine the impact of energy consumption on GHG emissions. Evaluation measures were Mean Squared Error (MSE), Mean Absolute Error (MAE), Mean Absolute Percentage Error (MAPE), Coefficient of Determination (R2) and Root Mean Squared Error (RMSE). The hybrid machine learning results show that long-term forecasting helps policymakers and environmental planners develop good mitigation strategies.

Hastomo et al. (2022) used GRU, LSTM and RMSprop optimisers to develop a model to simulate the carbon dioxide equivalent (CO2 eq) emissions from manure management, using 6 combinations of hidden layers. The research was conducted using a dataset from the Food and Agriculture Organisation (FAO) repository and took 15 epochs, with RMSE as the primary evaluation metric.

The study by Hamdan et al. (2023) utilised climate datasets from the National Oceanic and Atmospheric Administration (NOAA), taken about methane, carbon dioxide, nitrous oxide, and temperature as variables, and combined them with Earth angle data from the National Aeronautics and Space Administration (NASA). The study utilised an LSTM architecture to train the model over 25 epochs. It evaluated its performance using RMSE, achieving superior results compared to the baseline model in terms of accuracy and the effective management of complex temporal dependencies in climate data. In a different study, Sha et al. (2024) used the hybrid model (Bi-LSTM and GRU) to study the top greenhouse gas emitters. They have utilised the Adam optimiser with a learning rate of 0.001 and implemented a gating scheme to handle temporal variations in emissions better. The model was evaluated using MAPE, *R*^2^, and RMSE.

Ajala et al. (2025) forecast daily CO₂ emissions by comparing machine learning techniques (RF and Support Vector Regression) with deep learning methods (LSTM, Convolutional Neural Network (CNN), and GRU) and traditional models (ARIMA). RMSE, MAE, *R*^2^, and MAPE were used as evaluation metrics. Moreover, Hasan et al. (2025) used machine learning techniques, including RF, SVM, CNN, and LSTM, to forecast future greenhouse gas emissions trends. The study used data from various sources, including satellite, sensor, and drone observations, as well as manual inputs, to predict emissions across urban, industrial, agricultural, and natural areas. They increased the spatial resolution from 30 m to 10 m, reduced data reporting latency to 1 h, and found that LSTM outperformed other models.

McParland et al. (2024) developed and implemented a machine learning model to predict methane emissions from dairy cows. Milk yield, MIR spectra, milk composition, and days in milk (DIM) were used as model inputs, and fourfold cross-validation was performed during model development. The dataset from 2020 to 2022, with multiple breeds and crossbreds, was used to predict the emissions. The models used were neural networks (NN) and partial least squares regression (PLSR), and PLSR outperforms the NN model. In another study, Pence et al. (2024) developed a machine learning model, such as RF, KNN, Categorical Boosting (CatBoost), Light Gradient Boosting Machine (LGBM), Gradient Boosting (GB), and eXtreme Gradient Boosting Regressor (XGBR), to forecast N_2_O, CH_4_, and CO₂ emissions from dairy farms. The study develops two scenarios based on climate parameters and geographic structure, and uses an adaptive neuro-fuzzy inference system to predict GHG emissions. Scenario 1 included eight features (cattle population, agricultural area, humidity, wind speed, temperature, small ruminant, precipitation, and poultry population). In comparison, scenario 2 included 12 features, adding four from scenario 1: soil temperature at 5 cm, maximum temperature, minimum temperature, and sunshine duration. In both scenarios, CatBoost consistently outperformed the others, as shown by Sahraei et al. (2025) proposed using deep learning models, including LSTM, CNN, and Hybrid CNN-LSTM, to predict methane emissions from Holstein dairy cows based on feeding behaviour, feed intake, milk yield, and weather data. Sniffer technology was employed to measure methane emissions. The dataset was divided into four scenarios: scenario 1 (S1), which includes all features; scenario 2 (S2), with moderate and public features; scenario 3 (S3), containing only public features; and scenario 4 (S4), combining features from S2 and key rare features. LSTM performed best when all features were included, with an *R*^2^ of 0.88 and an MBE of 13.55 ppm, whereas CNN performed moderately due to its limited ability to capture long-term dependencies. Feeding-related data were the most critical predictors of methane emissions.

Bi and Neethirajan (2024) used XGBoost, LSTM, TCN, and Transformer models to predict methane emissions based on a dataset that included ERA5 meteorological data (temperature and wind speed), methane concentration from Sentinel-5P satellite (7 km × 7 km resolution), and seasonal cyclical features for dry and wet seasons, along with methane lag from 1 to 14. XGBoost outperformed other models, achieving an *R*^2^ of 0.7517 and an MAE of 4.69 ppb. SHAP analysis indicates that methane lag 1 was the strongest predictor, contributing 12.3 ppb, followed by the wet season at 8.7 ppb. Trend analysis reveals methane peaks in May during the wet season, reaching up to 200 ± 20 ppb, followed by a 6.8% decrease from the May peak to September, with levels of 1875 ± 15 ppb for Lamorde and 1890 ± 15 ppb for the cattle market. The study underestimates residuals, which can reach up to 50 ppb, and did not incorporate ground measurements for validation, relying instead on interpolation.

A study done by Solazzo et al. (2021) quantifies uncertainty using emission factors and activity data using IPCC (2006) default values, assuming log-normal distributions from the Emissions Database for Global Atmospheric Research (EDGAR) inventory. Nevertheless, Schulte et al. (2024) addressed the limitation in the study of Solazzo et al. (2021) by estimating uncertainty in the raw input data rather than relying on simplistic assumptions and correlations between variables, and by disaggregating common input data points by sampling from Dirichlet distributions (Schulte et al., 2024). Furthermore, Liu et al. (2025) improved the Monte Carlo method (MCM) by using Latin Hypercube sampling (LHS) rather than random sampling to quantify uncertainty. The study analyses the sources, calculates emission factors (mean, min, and max), and uses them to define assumptions. Each input was divided into non-overlapping equal-probability intervals based on the LHS. The improved MCM still relies on default values and literature data (Liu et al., 2025).

Moreover, Sykes et al. (2019) quantify and analyse uncertainty in emissions and identify the most influential sources of uncertainty for the beef system at the farm level. Use a Monte Carlo simulation with 10,000 runs to propagate uncertainty by applying probability distributions to emission factors and coefficients. Lognormal distribution was used for N₂O emission factors, while normal distributions were applied to enteric fermentation parameters for CH₄. Beta/PERT distributions were applied to energy, feed production, electricity, and fertiliser emissions. Tornado and scree plots were used to identify key uncertainty drivers in sensitivity analysis (Sykes et al., 2019).

In summary, the literature reveals three main gaps that motivate this study. First, while many studies have employed deep learning models (LSTM, GRU, BiLSTM) for GHG forecasting (Emami Javanmard and Ghaderi, 2022; Hastomo et al., 2022; Sha et al., 2024). Few have integrated both farm-level ground-based sensor data and remote sensing data. Studies that use satellite data alone (Bi and Neethirajan, 2024) or laboratory data alone (McParland et al., 2024) may lack the spatial or temporal resolution needed for farm-scale decision-making. Second, uncertainty quantification in existing GHG forecasting studies focused on aleatoric uncertainty (data noise) while neglecting epistemic uncertainty (model ignorance). Studies by Solazzo et al. (2021) and Schulte et al. (2024) Quantify uncertainty in emission inventories using Monte Carlo methods, but they do not distinguish among uncertainty types or propagate them into deep learning forecasts. Third, environmental factors such as humidity, temperature, and wind speed, which significantly influence gas dispersion and microbial processes, are often omitted or interpolated from distant stations (Sahraei et al., 2025).

This study proposes combining a Gated Recurrent Unit (GRU) model with uncertainty quantification to predict greenhouse gas emissions from ruminant livestock. It aims to fill three main gaps: (1) integrating ground-based sensor data with satellite-derived vegetation indices [normalised difference vegetation index (NDVI) and enhanced vegetation index (EVI)], land surface temperature (LST), and evapotranspiration (ET) to improve data quality and spatial coverage, thereby enabling a scalable and adaptive monitoring system for evolving farm conditions; (2) measuring both epistemic uncertainty with Monte Carlo dropout and aleatoric uncertainty with heteroscedastic Gaussian Negative Log-Likelihood to evaluate prediction confidence and variability; and (3) incorporating detailed on-site meteorological data (temperature, humidity, rainfall, wind speed) as additional inputs alongside remote sensing data. This combined approach advances farm-level GHG forecasting and offers clear, interpretable uncertainty estimates to support better decision-making.

**Figure 1:**
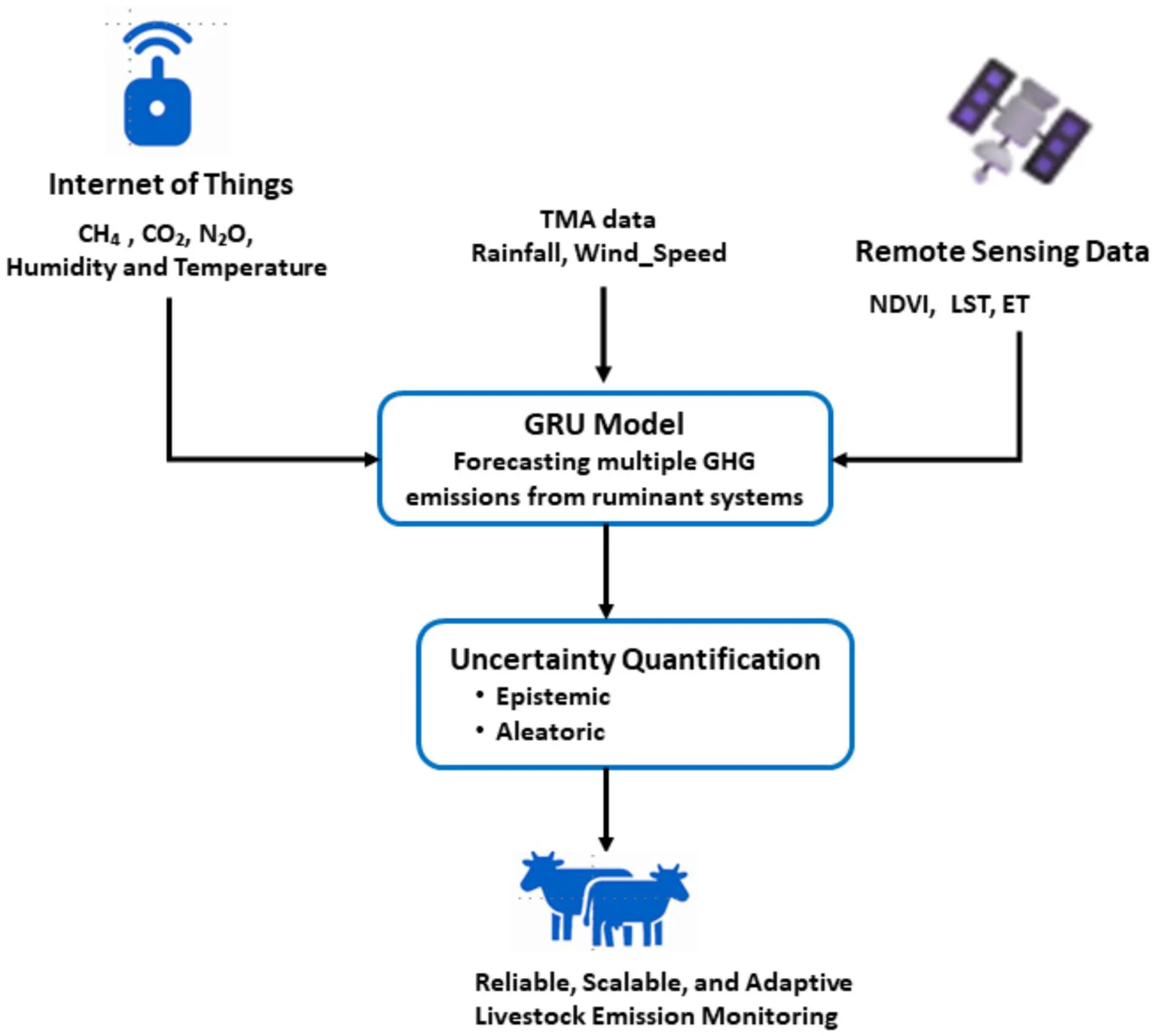
Conceptual framework of the proposed solution.

Thus, the objectives of this study are to (1) develop and compare the performance of the recurrent neural network (RNN) algorithm models and non-stationary time series models, and (2) quantify epistemic and aleatoric uncertainty for model reliability. Figure 1 illustrates the development and implementation of the proposed solution, which includes the necessary elements to support real-time multivariate forecasting of greenhouse gas concentrations from ruminants and to quantify uncertainty.

## 2 Methodology and model development

### 2.1 Study area

**Figure 2:**
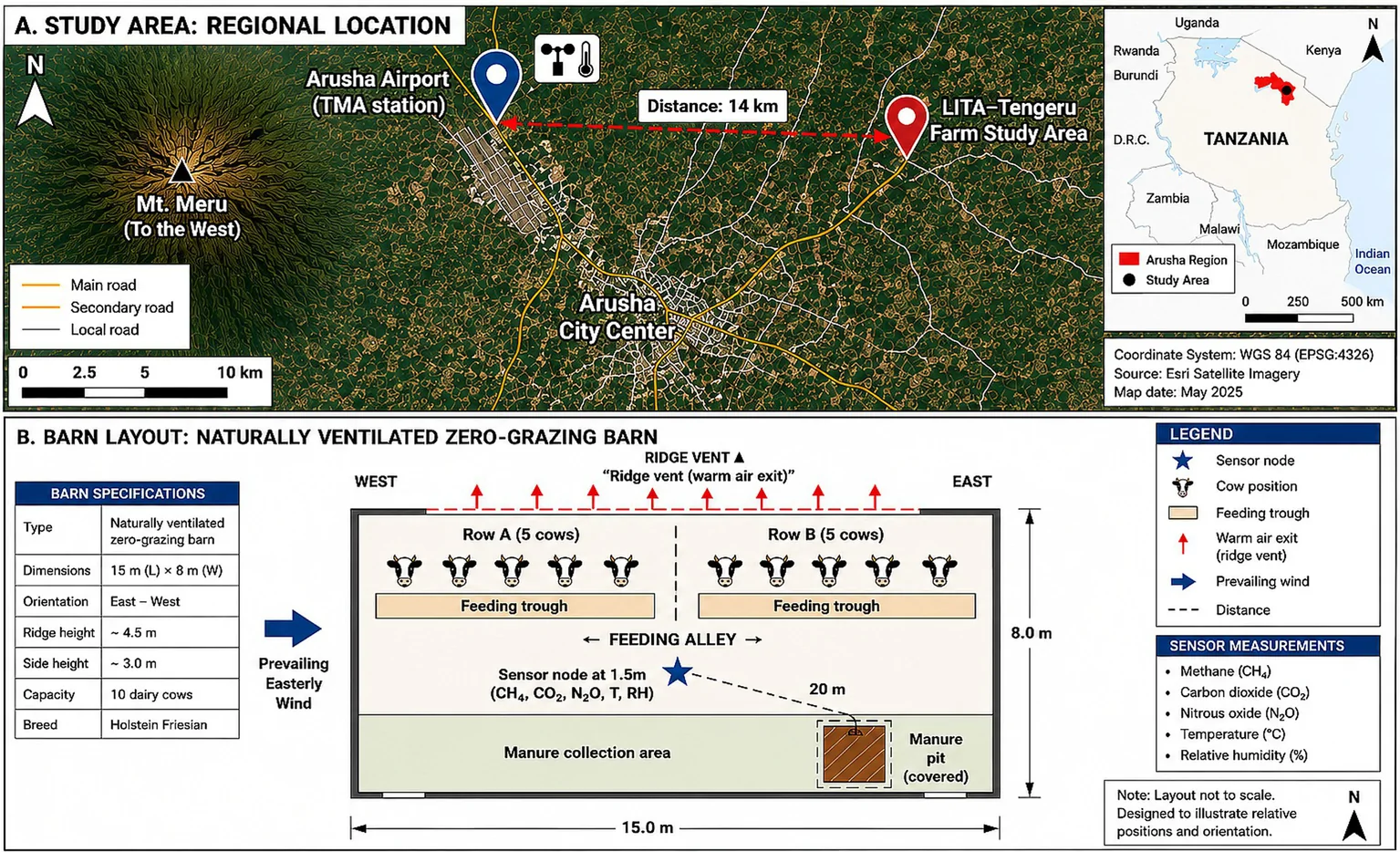
Study area and barn layout at the LITA–Tengeru dairy farm, Arusha, Tanzania. Panel **(A)** shows the regional location of the study site relative to Arusha Airport meteorological station (TMA station), and Arusha City Centre, including the 14 km distance between the meteorological station and the study farm. The inset map indicates the location of the study area within the Arusha Region, northern Tanzania. Panel **(B)** illustrates the naturally ventilated zero-grazing barn layout, including barn dimensions and orientation, cow positions, feeding troughs, manure collection area, manure pit location, sensor node placement at cow breathing height (1.5 m), ridge vent configuration, and prevailing easterly wind direction. Environmental measurements collected at the sensor node included methane (CH₄), carbon dioxide (CO₂), nitrous oxide (N₂O), temperature, and relative humidity. Satellite imagery source: ESRI World Imagery; coordinate reference system: WGS 84 (EPSG:4326).

This study was carried out in the Arumeru district in the Arusha region, part of Tanzania’s northern zone, which includes three regions: Arusha, Manyara, and Kilimanjaro. The LITA-Tengeru campus was chosen because it represents the northern livestock zone. A census-based sampling approach was used, including all cows on the farm to ensure thorough coverage and prevent bias (Creswell and Creswell, 2017; Kothari, 2004). Dairy cattle were specifically targeted since they are a major source of GHG emissions, especially methane from enteric fermentation (Ghassemi Nejad et al., 2024). Sensors were installed at the LITA-Tengeru campus at 27°S latitude, 37°E longitude, and 1,250 meters above sea level. Figure 2 illustrates the study area.

### 2.2 Data collection and preprocessing

#### 2.2.1 Ground-based sensors measurements (primary dataset)

A total of 1,270,080 concentration observations of nitrous oxide (N₂O) parts per billion (PPB), methane (CH₄) (PPB), and carbon dioxide (CO₂) parts per million (PPM) were collected on site via *in situ* sensing. Weather features, including temperature (°C) and humidity (%), were collected on-site. An on-site dataset was collected from January 2023 to May 2025 at one-minute intervals. Data were aggregated to an hourly interval for data management, yielding 21,168 data points. The GHG, temperature and humidity sensor node was positioned at approximately 1.5 m above ground level within the feeding alley, corresponding to the typical animal breathing zone and the primary area of cattle occupancy. Temperature and relative humidity were measured using a DHT22 environmental sensor integrated into the same sensor node. The DHT22 sensor was mounted on the same support structure at a height of 1.5 m, adjacent to the MQ-4, MQ-135, and TDS-0131 sensors. This co-location ensured that temperature and humidity measurements represented the same micro-environment as the gas concentration measurements and supported compensation for environmental sensor cross-sensitivities during calibration.

This placement was intended to capture ambient greenhouse gas concentration dynamics influenced by enteric fermentation, manure decomposition, and animal respiration under naturally ventilated conditions. Similar placement strategies have been reported in previous livestock air-quality and greenhouse gas monitoring studies in naturally ventilated dairy systems (D’Urso et al., 2025; McGinn and Beauchemin, 2012). As the barn operated under open or naturally ventilated conditions, measured concentrations were influenced by airflow patterns, wind direction, and atmospheric mixing. The sensor was located approximately 10 m from the cattle feeding and resting area and approximately 20 m from the manure collection pit. Under prevailing easterly winds common in the Arusha region, measured concentrations likely reflected a stronger influence from cattle-associated emissions, whereas westerly winds may increase the relative contribution of manure-derived CH₄ and N₂O. Consequently, the measurements should be interpreted as mixed ambient concentration signals rather than direct source-specific emission fluxes, as shown in Figure 2. No mobile sensors were used; data collection was confined to this fixed site.

#### 2.2.2 Sensor calibration and data quality

Sensor calibration was performed at Droid Technologies Limited using certified reference gases. It was carried out under controlled environmental conditions with temperatures ranging from 5 to 30 °C and relative humidity between 40 and 80%. This range was chosen to mirror typical ambient conditions while staying within the stability limits of metal-oxide-semiconductor (MOS) sensors. Studies have indicated that MOS sensor responses are significantly affected by temperature and humidity, with greater drift, hysteresis, and cross-sensitivity at higher humidity levels and at temperature extremes (Barsan and Weimar, 2001; Hong et al., 2023; Wang et al., 2010). Therefore, the calibration range was selected to represent a controlled set of conditions in which sensor responses are more stable and reproducible, yet still reflect the variability seen in real-world applications. Response drift was corrected using nonlinear regression models that included interaction terms between temperature and relative humidity.

The general structure of the calibration model was expressed as a function of the raw sensor signal and environmental covariates: C_corrected = f(S_raw, T, RH), where: C_corrected = calibrated (or corrected) gas concentration, S_raw = raw sensor output, T = ambient temperature, and RH = relative humidity (Hong et al., 2023; Kim et al., 2021; Masson et al., 2015; Taguem et al., 2021). Interaction components were retained in the final specification where they improved fit and reduced systematic bias associated with environmental variability. Calibration accuracy was evaluated against reference gas concentrations using the coefficient of determination (*R*^2^), Root Mean Square Error (RMSE), Mean Absolute Error (MAE), and mean relative percentage error.

#### 2.2.3 Secondary dataset

Rainfall and wind speed data were collected from the Tanzania Meteorological Agency (TMA) at Arusha Airport, about 14 km from the LITA-Tengeru campus, from January 2023 to May 2025 and were used as exogenous meteorological inputs. Vegetation indices, specifically the Normalized Difference Vegetation Index (NDVI) and the Enhanced Vegetation Index (EVI), were derived from Sentinel-2 MSI imagery at 10–20 m resolution, with new values available roughly every 5 days. Land surface temperature (LST) and evapotranspiration (ET), on the other hand, were derived from the MODIS MOD11A2 and MYD11A2 products, which are released only every 8 days. Because NDVI and EVI tend to change gradually, linear interpolation was used to bridge the gaps between satellite passes and produce a continuous hourly predictor series. LST and ET were treated differently: both varied sharply over a 24-h cycle, so interpolating hour-by-hour between eight-day snapshots would have produced physically unrealistic values, particularly at night, where a simple linear fit would drag values upward towards daily highs. Rather than forcing that interpolation, the last observed satellite value was held constant until the next acquisition date.

#### 2.2.4 Animal information

The study involved 10 lactating Holstein Friesian cattle kept in a zero-grazing barn at the LITA-Tengeru campus. The barn, roughly 15 m by 8 m, featured a gable roof and an open front to promote natural airflow. Cattle were arranged in two rows of five, with individual stalls 1.2 m wide for each animal. Their weights ranged from 450 kg to 650 kg, and average milk production was between 12 and 15 litres per day per cow. Feeding was ad libitum using a cut-and-carry system. Fresh feed was provided twice daily (morning and afternoon), but cattle consumed feed continuously throughout the day with no fixed feeding schedule. Milking was conducted twice daily at 07:00–08:00 and 16:00–18:00. Manure was removed twice daily at 08:00 and 17:00 and stored in a nearby pit about 20 meters from the sensor station. The feed consisted of elephant grass (*Pennisetum purpureum*), Rhodes grass (*Chloris gayana*), some legumes such as Desmodium spp., small amounts of concentrates (maize bran, sunflower seed cake), and mineral supplements, including table salt and lime. Greenhouse gas emissions came from three main sources: (1) enteric fermentation producing methane during and after feeding; (2) manure decomposition releasing methane and nitrous oxide from the collection area and storage pit; and (3) animal respiration emitting carbon dioxide. A sensor was installed at a height of 1.5 m in the feeding alley to monitor gases from all three sources.

### 2.3 Model development, training, and testing

Keras was used to develop the model with a TensorFlow backend. In total, 80% of the dataset was used for training, and the remaining 20% for testing and validation. The Len() function was used to split the training and testing datasets reproducibly, allowing quantification of dataset size and the number of samples available in the sequence, and enabling the window to slide through the time series. The study employed various hyperparameter tuning methods with Optuna (Akiba et al., 2019). The hyperparameters included the number of units (150 and 100), batch sizes, dropout rate (0.1, 0.2, 0.1, 0.2, 0.1, 0.2, 0.3, and 0.4), learning rates [1e-4, 1e-3], epochs (50, 50,100), optimisers (Adam and RMSprop), recurrent dropout, and activation functions (tanh (state update), sigmoid (gates), tanh (cell state)). This was done to ensure the model achieves optimal, reliable results, which are crucial for informed decision-making by policymakers and livestock stakeholders. Optuna conducted 50 trials per gas species, selecting configurations that minimised validation RMSE within the 5-fold rolling cross-validation framework.

To prevent bias in some features and ensure consistency in all features, the study normalised the dataset by min-max normalisation (Siami-Namini et al., 2019). Using ARIMAX and SARIMAX to derive models, the Akaike Information Criterion (AIC) and Bayesian Information Criterion (BIC) were used to assess the trade-off between complexity and goodness of fit, providing suitable statistical foundations for time series modelling.

#### 2.3.1 Forecasting setup

The forecasting problem was formulated as a direct, single-step, multivariate time-series problem. The multivariate predictor vector at time 𝑡 t is denoted as $X_{t}\in R^{d}$ , where 𝑑 represents the number of predictors retained after fold-wise feature selection. The forecasting task is formulated as learning a mapping function, as shown in Equation 1.


$$f_{\theta }(X_{t-L+1},X_{t-L+2},\ldots ,X_{t})\to y_{t+h}$$ (1)

where *L* represents the lookback window, and *h* indicates the forecasting horizon. In this study, *L* = 24 h and *h* = 1. The direct forecasting method, where each prediction relies only on previously observed inputs, is based on traditional time-series forecasting techniques to prevent the accumulation of errors (Ben Taieb et al., 2012). The GRU model learns to map the past 24-h sequence of environmental and concentration predictors to the next-hour concentration value. The probabilistic formulation enables the network to estimate both the conditional mean and the conditional variance of the predicted concentration.

The 24-h lookback window was chosen to capture daily patterns in greenhouse gas levels in controlled zero-grazing systems, where concentration trends typically follow daily cycles influenced by feeding schedules, barn ventilation, and microclimate factors. For each prediction at time t, the model takes the ordered sequence (*X*_t-23_……., *X*_t_), helping it learns short-term dependencies and recurring daily patterns. Using a one-step-ahead forecast horizon guarantees strict chronological causality and reduces error buildup. A direct forecasting method is applied to avoid cumulative errors, with each prediction relying only on past observed inputs, not on previous predictions fed back into the model. Separate models were trained for nitrous oxide, methane, and carbon dioxide, allowing independent parameter estimation and uncertainty analysis for each gas. During training, the input tensor is shaped (batch size, 24, *d*) to preserve temporal order and support reproducibility.

Separate models were trained for methane, carbon dioxide, and nitrous oxide. The output layer is implemented in a dual-head fashion, producing the conditional mean simultaneously, as in Equation 2. The variance term is exponentiated during inference to ensure positivity. Prediction intervals are then obtained as specified in the uncertainty framework, as shown in Equation 3.


$$\mu_{t},\log(\sigma_{t}^{2})\;$$ (2)

$$\sigma_{t}^{2}\exp(\log(\sigma_{t}^{2}))\;$$ (3)

This dual-head architecture, which outputs the conditional mean and log variance, is based on the heteroscedastic Gaussian likelihood framework introduced by Nix and Weigend (1994) for neural network uncertainty estimation. Recent progress has shown that such architectures are now common for simultaneously learning the mean and variance in regression tasks (Immer et al., 2023).

#### 2.3.2 Seasonal naïve model

The Seasonal Naive model was implemented as a baseline model to compare the performance of the RNN model. The approach assumes that future values will repeat the most frequently observed values in the same 24-h seasonal period. This model was selected because it provides an interpretable baseline reference and a transparent point of comparison that reflects robust seasonal and diurnal patterns in GHG concentrations. This model does not require parameter estimation, making it robust and computationally efficient. The formula for the seasonal naive model is illustrated in Equation 4.


$$\hat{y}(t+h)=y(t+h-s)\;$$ (4)

where *ŷ*(*t* + *h*) is the forecast for horizon *h*, *y*(*t* + *h-s*) is the observed value from the previous seasonal cycle, and *s* is the seasonal period (e.g., *s* = 24 h).

#### 2.3.3 Autoregressive integrated moving average with exogenous variables (ARIMAX)

The study implemented the ARIMAX model to include external predictors while maintaining linear temporal dynamics as shown in Equation 5. The Augmented Dickey-Fuller (ADF) test was employed to evaluate stationarity in the time series data. Its null hypothesis states that a unit root exists, indicating non-stationarity. A null hypothesis is rejected if the test statistic falls below the critical value at the 5% significance level, indicating that the series is stationary (Dil, 2025; Ivanovski and Ivanovska, 2024; Maitra and Politis, 2024; Said and Dickey, 1984). When non-stationarity was identified (*p* > 0.05), first-order differencing was performed to achieve stationarity prior to model fitting.


$$\begin{array}{l} y(t)=c+\varphi^{1}y(t-1)+\ldots +\mathrm{\varphi py}(t-p)+\beta^{1}x^{1}(t)+\ldots \\ +\text{βkxk}(t)+\theta^{1}\varepsilon (t-1)+\ldots +\mathrm{\theta q\varepsilon}(t-q)+\varepsilon (t) \end{array}$$ (5)

Where *y*(*t*) is the differenced target series, *x*(*t*) are exogenous variables, *φ* and *θ* are AR and MA coefficients, *β* are regression coefficients, and *ε*(*t*) is white noise.

#### 2.3.4 Seasonal autoregressive integrated moving average with exogenous variables (SARIMAX)

The study employs the SARIMAX model to account for the seasonal and diurnal periodicity in GHG concentrations. The model incorporates exogenous variables. Seasonal differencing was applied when necessary to preserve stationarity. The model was defined by Equation 6.


$$\begin{array}{l} y(t)=c+\mathrm{\varphi y}(t-1)+\mathrm{\Phi y}(t-s)+\mathrm{\beta X}(t)\\ +\theta \varepsilon (t-1)+\Theta \varepsilon (t-s)+\varepsilon (t)\;\;\;\; \end{array}$$ (6)

Where *s* is the seasonal period, *X(t)* denotes exogenous variables, *φ* and *Φ* are non-seasonal and seasonal AR terms, *θ* and *Θ* are MA terms, and *ε*(*t*) is white noise.

#### 2.3.5 Long short-term memory

The long short-term memory (LSTM) algorithm is a type of deep learning algorithm that builds on the recurrent neural network (RNN) (Hochreiter and Schmidhuber, 1997; Houssein et al., 2025). The algorithm has three gates: the input gate, the forget gate, and the output gate. These three gates are considered a storage memory (cell) in the LSTM algorithm. A time-recursive neural network can learn from data over the long term and is suitable for processing and forecasting events with specific intervals and delays in time series. The LSTM model architecture consists of five layers. The first layer is an LSTM layer with 150 units, which returns sequences of shape (None, 24, 150) and contains 96,600 trainable parameters. This is followed by a dropout layer with a rate of 0.4 for regularisation, which adds no parameters. The second LSTM layer also contains 150 units but returns only the final output of shape (None, 150), contributing 180,600 parameters. Another dropout layer follows, again with a 0.4 dropout rate. Finally, a dense output layer with one unit produces the next-hour concentration prediction, adding 151 parameters. In total, the model has 277,351 trainable parameters, equivalent to 1.06 MB of memory. In the output shape notation, none denotes the variable batch size (set to 32 in this study), 24 is the temporal lookback window (in hours), and 150 is the number of LSTM units in the layer. No non-trainable parameters are present. Hyperparameters such as the optimiser, batch size, and activation functions vary by gas species, as detailed in Table 1.

**Table 1:** Hyperparameter values and choices used during training of the LSTM model.

Gas	Best parameters				
	Unit	Dropout rate	Optimizer	Batch size	Activation functions
Carbon dioxide	150	0.4	adam	32	tanh (cell state), sigmoid (gates)
Methane	150	0.4	rmsprop	32	tanh (cell state), sigmoid (gates)
Nitrous oxide	150	0.2	adam	32	tanh (cell state), sigmoid (gates)

#### 2.3.6 Gated recurrent unit

A Gated Recurrent Unit (GRU) is a variant of the RNN architecture designed to model sequential data by selectively remembering or forgetting information over time. The GRU architecture is more efficient and has fewer learnable parameters (Cho et al., 2014). GRU also uses the update and reset gates to address vanishing and exploding gradients. The architecture of the GRU model comprises five layers. The first is a GRU layer with 150 units, outputting sequences of shape (None, 24, 150) and containing 72,900 trainable parameters. This is followed by a dropout layer with a rate between 0.2 and 0.3 (depending on the gas species) for regularisation, which adds no parameters. The second GRU layer also has 150 units but outputs only the final state of shape (None, 150), with 135,900 parameters. Another dropout layer with a similar rate follows. The final layer is a dense layer of neurons that predicts the next hour’s concentration, adding 151 parameters. Overall, the model has 208,951 trainable parameters and occupies about 816.21 KB of memory. In the shape notation, none indicates the variable batch size (set to 32 here), 24 signifies the temporal lookback period in hours, and 150 denotes the number of units in each GRU layer. There are no non-trainable parameters. Details on hyperparameters are available in Table 2.

**Table 2:** Hyperparameter values and choices used during training of the GRU model.

Gas	Best parameters				
	Unit	Dropout rate	Optimizer	Batch size	Activation functions
Carbon dioxide	150	0.3	adam	32	tanh (state update), sigmoid (gates)
Methane	150	0.2	adam	32	tanh (state update), sigmoid (gates)
Nitrous oxide	150	0.3	adam	32	tanh (state update), sigmoid (gates)

#### 2.3.7 Bidirectional (BiLSTM)

Bidirectional LSTM enables additional training by traversing the sequence in both the forward (left-to-right) and backward (right-to-left) directions, then concatenating their hidden states at each time step, thereby enhancing accuracy (Graves et al., 2005; Sha et al., 2024). It is beneficial to interpret current emissions in light of historical trends. The BiLSTM model consists of five layers. The initial layer is a BiLSTM with 150 units, producing output sequences with shape (None, 24, 150) and containing 72,900 trainable parameters. Next, a dropout layer with a rate between 0.2 and 0.3 (depending on the gas species) is applied for regularisation, which does not add parameters. The second BiLSTM layer also has 150 units but outputs only the final state, with a shape of (None, 150) and 135,900 parameters. Another dropout layer with a similar rate follows. The final layer is a dense neural network layer that predicts the concentration for the next hour, with 151 parameters. The model has 208,951 trainable parameters and uses approximately 816.21 KB of memory. In the shape notation, none represents the variable batch size (set here to 32), 24 indicates the lookback period in hours, and 150 is the number of units per BiLSTM layer. The model has no non-trainable parameters. Hyperparameter details are provided in Table 3.

**Table 3:** Hyperparameter values and choices used during training of the BiLSTM model.

Gas	Best parameters				
	Unit	Dropout rate	Optimizer	Batch size	Activation functions
Carbon dioxide	100	0.3	adam	32	tanh (cell state), sigmoid (gates)
Methane	150	0.3	adam	32	tanh (cell state), sigmoid (gates)
Nitrous oxide	150	0.2	adam	32	tanh (cell state), sigmoid (gates)

A two-layer model was chosen to capture temporal dependencies without overfitting. For methane, 150 units were selected for BiLSTM, LSTM and GRU due to its higher temporal variability. For nitrous oxide and carbon dioxide, 100 units were used, as they are more stable. Dropout rates of 0.2–0.4 were applied after each recurrent layer: higher rates (0.3–0.4) were used for CH₄ to regularise its greater complexity, while lower rates (0.2) were used for N₂O (Soomro, 2022; Tirchas et al., 2024).

### 2.4 Feature engineering, selection, and evaluation

The study employed various feature engineering techniques to capture temporal dependencies and environmental effects on GHG levels. Lag features were created to predict concentration values based on previous time steps (*t*-1, *t*-2,…, *t*-24). A lag variable helped account for transport delay between the emission source and the sensor, as well as temporal autocorrelation (Vitale et al., 2024). Rolling statistics, like the 24-h rolling mean (roll24_mean) and variance, were computed using a backwards-looking window that included the current observation and the previous 23 h, reducing short-term noise while maintaining trends (Derkacheva et al., 2020). Long-term concentration trends were extracted with a Hodrick-Prescott filter (*λ* = 1,600 for hourly data) to distinguish seasonal and annual changes from short-term fluctuations (Hodrick and Prescott, 1997; Mohamed and Mohammed, 2021). Vegetation indices (NDVI and EVI) served as proxies for feed availability and quality, with NDVI validated as an indicator of forage quality and crude protein in African savannas, and EVI providing better sensitivity under high-biomass conditions (Tong et al., 2019). Cyclical encodings involved transforming day of year and hour of day using sine and cosine functions to preserve their circular nature, improving forecast accuracy (Elloumi et al., 2025).

Feature selection was conducted independently in each training fold during rolling cross-validation to avoid information leakage. Initially, predictors with weak associations (absolute Pearson correlation ≤ 0.1) were filtered out, and highly collinear variables (|*r*| > 0.85) were removed to reduce redundancy. Following this, two ranking methods were employed: F-regression to evaluate univariate feature-target correlations, and Recursive Feature Elimination (RFE) using a lightweight GRU-based surrogate model. This surrogate, a simplified single-layer GRU trained specifically to determine feature importance for elimination, differs from the final production GRU (Section 2.5) in architecture (64 vs. 150 hidden units) and purpose (ranking vs. forecasting). The importance rankings from each method varied across the training set of each fold. Scores were normalised within each fold using min-max scaling. The normalised importance scores from correlation filtering, F-regression, and RFE were then averaged to produce a combined importance score for each feature, with relative importance represented by Equation 7 (Liu et al., 2021; Tumay et al., 2023).


$$\text{Importanc}e_{i}(\backslash \%)=\frac{I_{i}}{\sum_{j=1}^{k}I_{j}}\times 100\;$$ (7)

The reported values are the importance values of the average normalised ensemble score, computed over five rolling validation folds. A feature was ranked in the top 10 in at least three of the five folds. The consistency of rankings was assessed by the standard deviation of the importance scores across folds, providing a quantitative measure of selection consistency when time is resampled (Bertolini and Finch, 2024; Łukaszuk et al., 2024).

Although NDVI and EVI were strongly correlated within the dataset, they represent distinct vegetation characteristics. NDVI primarily reflects vegetation vigour and chlorophyll activity, whereas EVI is less sensitive to atmospheric effects and saturation at high biomass levels. Both variables were retained because recursive feature elimination and fold-wise feature selection identified independent predictive contributions for different greenhouse gases. Similarly, satellite-derived land surface temperature (LST) and on-site air temperature represent different physical processes. LST reflects surface radiative heating conditions associated with soil thermal dynamics and evapotranspiration, whereas on-site air temperature reflects near-surface atmospheric conditions directly affecting animal physiology and enteric fermentation processes. Variance inflation factor analysis and recursive feature elimination were used to minimise multicollinearity-related instability during model development. Although NDVI and EVI, along with LST and air temperature, had strong pairwise correlations (|*r*| up to 0.85), multicollinearity is of lower concern in neural networks than in multiple regression models, in which coefficient estimation and interpretation can be negatively impacted by variance inflation (Dormann et al., 2013). Nevertheless, highly correlated variables can lead to feature redundancy and unnecessary model complexity (Ayinde et al., 2019), thereby increasing the risk of overfitting (Yoon and Hwang, 2017).

### 2.5 Uncertainty quantification

The predictive reliability was determined using a framework that compares parameter uncertainty to data-driven variability as a separate quantity. The modelling approach isolates the uncertainty that can be ascribed to a lack of knowledge about the learned weights (epistemic) and that arising from the variability inherent in the very process of observation (aleatoric). Within this structure, the GRU network was configured to have two output heads. One head estimates the conditional mean (*μ*) while the other estimates the logarithm of the conditional variance (log *σ*^2^). The variance component is exponentiated during inference to ensure that *σ*^2^ is always positive and numerically stable. Aleatoric uncertainty learned under a heteroscedastic Gaussian likelihood formulation. Training minimised the Gaussian Negative Log-Likelihood (NLL), which is elaborated in Equation 8.


$$L_{\mathrm{NLL}}=(1/2)\log(\sigma_{t}^{2})+({\left(y_{t}-\mu_{t}\right)}^{2})/(2\sigma_{t}^{2})\;\;$$ (8)

where *y* represents the observed concentration, *μ* the predicted mean, and *σ*^2^ the predicted variance. This objective function penalises inaccurate predictions while simultaneously calibrating dispersion, thereby discouraging variance collapse or artificial inflation.

Epistemic uncertainty was estimated by Monte Carlo Dropout. Dropout layers remained active during inference, and 100 stochastic forward passes were executed per prediction (Gal and Ghahramani, 2016). Convergence diagnostics showed that the predictive variance stabilised after about 80 iterations, with <1% change after 100 iterations. Therefore, the choice of *N* = 100 balances statistical convergence and computational burden. Equation 9 shows how the variance across several stochastic forward passes was computed; Equation 10 was used to compute the mean prediction over the *N* stochastic passes; and the total predictive variance was computed as elaborated in Equation 11


$$\sigma_{\text{epistemic}}^{2}=\frac{1}{N}\sum_{i=1}^{N}{\left(\hat{y_{t}^{\left(i\right)}}-\bar{y_{t}}\right)}^{2}\;\;$$ (9)

$$\bar{y_{t}}=\frac{1}{N}\sum_{i=1}^{N}\hat{y_{t}^{\left(i\right)}}$$ (10)

$$\sigma_{\text{total}}^{2}=\sigma_{\text{epistemic}}^{2}+\sigma_{\text{aleatoric}}^{2}$$ (11)

Assuming a Gaussian distribution, 95% prediction intervals were calculated as shown in Equation 12.


$$\hat{y_{t}}\pm 1.96\sigma_{t}$$ (12)

Prediction Interval Coverage Probability (PICP_95_) measures the empirical proportion of observations within the nominal 95% prediction interval. A perfectly calibrated model should have PICP close to 0.95. This technique has been established as a reliable metric for validating the calibration of prediction uncertainty (Zheng et al., 2021). Negative Log-Likelihood (NLL) is a scoring rule that evaluates both sharpness (concentration) and calibration (accuracy) for predictive distributions. Lower values indicate better probabilistic predictions (Hastie et al., 2009). Error-to-Uncertainty Ratio (EUR) is the ratio of absolute prediction error to predictive standard deviation. Values near 1 indicate well-calibrated uncertainty, >1 suggest underestimation of uncertainty, while values <1 suggest overestimation (ISO and OIML, 1993). The formula for EUR is shown in Equation 13.


$$\mathrm{EUR}=\frac{|y_{t}-\hat{y_{t}}|}{\sigma_{t}}\;$$ (13)

### 2.6 Performance evaluation metrics

The model was evaluated using the Coefficient of Determination (*R*^2^), Mean Absolute Error (MAE), and Root Mean Square Error (RMSE) metrics. The formulas for these metrics are shown in Equations 14–16.


$$R^{2}=1-\frac{\sum_{i=1}^{n}{\left(y_{i}-\hat{y_{i}}\right)}^{2}}{\sum_{i=1}^{n}{\left(y_{i}-\bar{y}\right)}^{2}}\;\;$$ (14)

$$\mathrm{MAE}=\frac{1}{n}\sum_{i=1}^{n}|y_{i}-\hat{y_{i}}|\;$$ (15)

$$\begin{array}{c} \text{RMSE}=\sqrt{\frac{1}{n}\sum_{i=1}^{n}{\left(y_{i}-\hat{y_{i}}\right)}^{2}}\;\;\; \end{array}$$ (16)

Where $y_{i}$ are the actual values, *ŷ* i are the predicted values, and n is the total number of observations. An *R*^2^ represents the proportion of the variance in the dependent variable that is predictable from the independent variables. An R_2_ of 1 represents perfect prediction, while an R_2_ of 0 is suggestive of the model not performing any better than the mean. RMSE is used to measure the distance between actual and predicted values. The metric squares the difference to eliminate negative signs and place greater weight on substantial differences. The lower the value of RMSE, the better we have fitted the model to the data. MAE is an evaluation metric that computes the mean absolute difference between actual and model-predicted values, which measures the average magnitude of error directly. This statistic is not as prone to extreme outliers and gives a more correct picture of the average level of error (Hodson, 2022).

### 2.7 Model validation and strictly causal cross-validation protocol

A rolling-origin cross-validation approach with five chronological folds was employed to ensure strict causal evaluation. In each fold, the training period precedes the validation period, ensuring that validation data always follow the training data. This method simulates real-world forecasting scenarios and prevents data leakage from future information into the model training. Before model development, preprocessing operations, such as handling missing values with interpolation, min-max normalisation, and lag and rolling statistics, were applied to the training and validation sets. Feature selection was then performed independently in each fold on the training dataset, followed by hyperparameter optimisation on the training set. The strictly causal evaluation protocol, as adopted from Bergmeir et al. (2018), Bergmeir and Benítez (2012), and Cerqueira et al. (2020), prevents temporal leakage and yields unbiased estimates of generalisation.

### 2.8 Evaluation protocol

The study employs a two-stage evaluation process to ensure an unbiased assessment of performance. In Stage 1, the dataset was divided chronologically into an 80% development set (January 2023–October 2024) and a 20% independent holdout test set (November 2024–May 2025). The holdout set was not used during model development, cross-validation, hyperparameter tuning or feature selection. Step two: a fivefold rolling cross-validation was performed on 80% of the model development data. This structure guarantees an unbiased evaluation and prevents data leakage (Bergmeir et al., 2018).

Traditional significance tests, such as paired *t*-tests, are avoided because autocorrelation in time-series data violates the assumption of independence and inflates type I error rates (Diebold and Mariano, 2002). Instead, performance is assessed using fold-level statistics from cross-validation, with the mean and standard deviation across folds providing a stable measure of model robustness.

## 3 Results

### 3.1 Statistical description of the dataset

The data includes 21,168 hourly data points between January 2023 and May 2025. The dataset range reflects the realism of atmospheric conditions and seasonal variation, providing a reliable basis for time series modelling, as shown in Table 4.

**Table 4:** Statistical summary for the variables.

Variable	Min	Max	Mean	Std
Temperature	15.76	33.83	24.69	3
Relative humidity	23.19	97.68	52.4	16.95
Methane (CH₄)	1868.34	1931.57	1902.23	15.33
Carbon dioxide (CO₂)	389.57	429.47	411.22	8.19
Nitrous oxide (N₂O)	314.23	346.05	331.85	10.59
Wind speed	0.14	7.88	3.8	1.36
NDVI	0.1	0.37	0.24	0.06
EVI	0.15	0.54	0.31	0.12
Land surface temperature	20.66	36.61	27.51	3.66
Rainfall (mm)	0	0.056	0.0013	0.003

### 3.2 Sensor calibration performance

**Figure 3:**
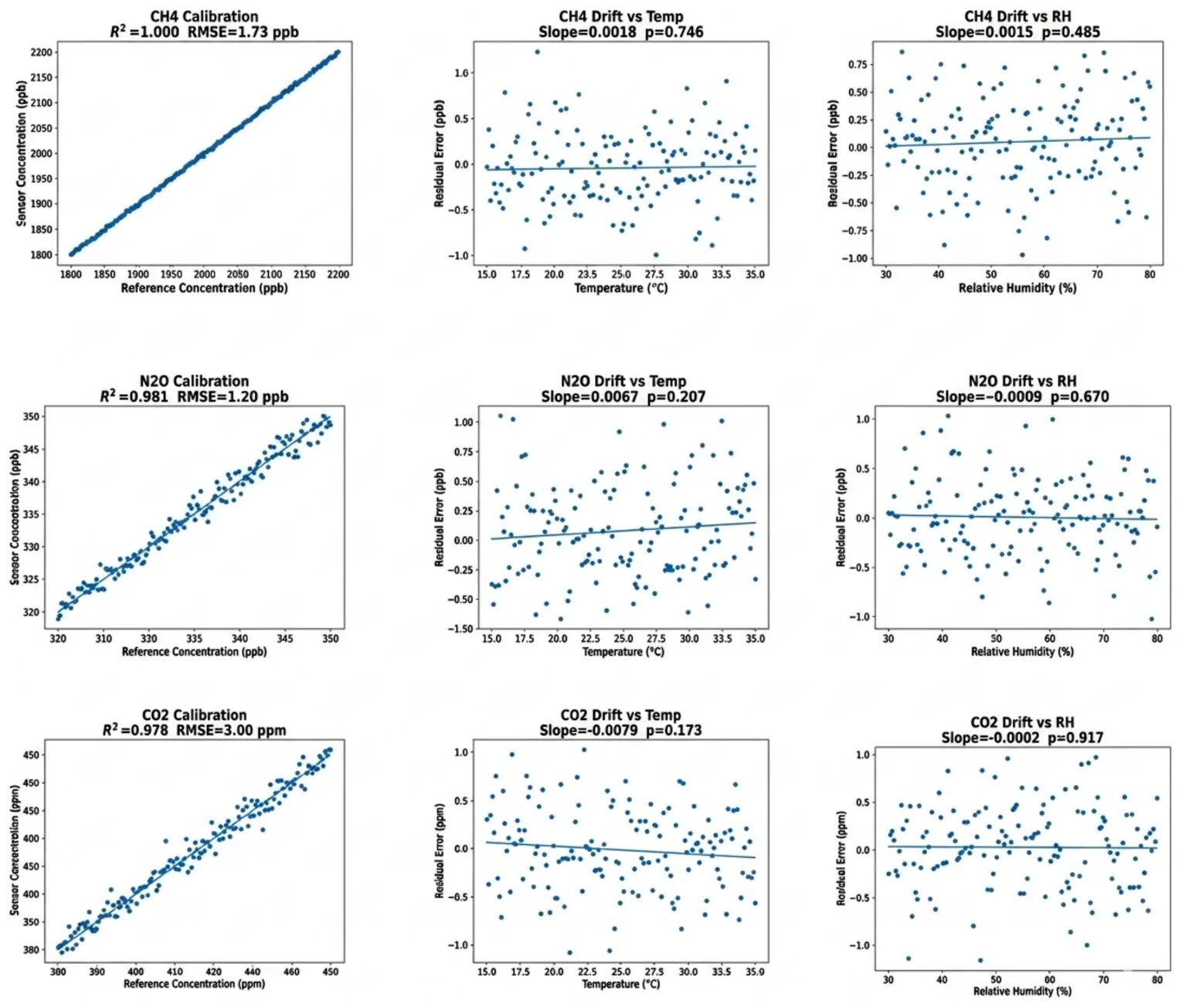
Sensor calibration and environmental stability validation. Top panel: Methane (CH₄) calibration showing reference vs. sensor readings (*R*^2^ = 0.98, RMSE = 1.84 ppb, mean relative error < 1%). Middle panel: Carbon dioxide (CO₂) calibration (*R*^2^ = 0.97, RMSE = 2.94 ppm, mean relative error < 1%). Bottom panel: Nitrous oxide (N₂O) calibration (*R*^2^ = 0.98, RMSE = 1.27 ppb, mean relative error < 1%). Calibration was performed at three concentration levels (low, medium, high) under controlled temperature (15–35 °C) and relative humidity (30–80% RH). No systematic drift was observed across the environmental range.

The sensor’s behaviour within the environmental range was validated in the laboratory, confirming its stability. The performance of all devices was strong for nonlinear calibration (*R*^2^ ≥ 0.971), with a relative mean error of less than 1%. Methane, carbon dioxide, and nitrous oxide sensors gave RMSE of 1.84 ppb, 2.94 ppm, and 1.27 ppb, respectively. The drift analysis was conducted under a control temperature of (15 °C–35 °C) and (30–80 %RH) for relative humidity. Systematic drift was not found in temperature or humidity gradients. These results justify the dataset’s suitability for downstream modelling and uncertainty quantification, as shown in Figure 3.

### 3.3 Feature engineering and selection

**Figure 4:**
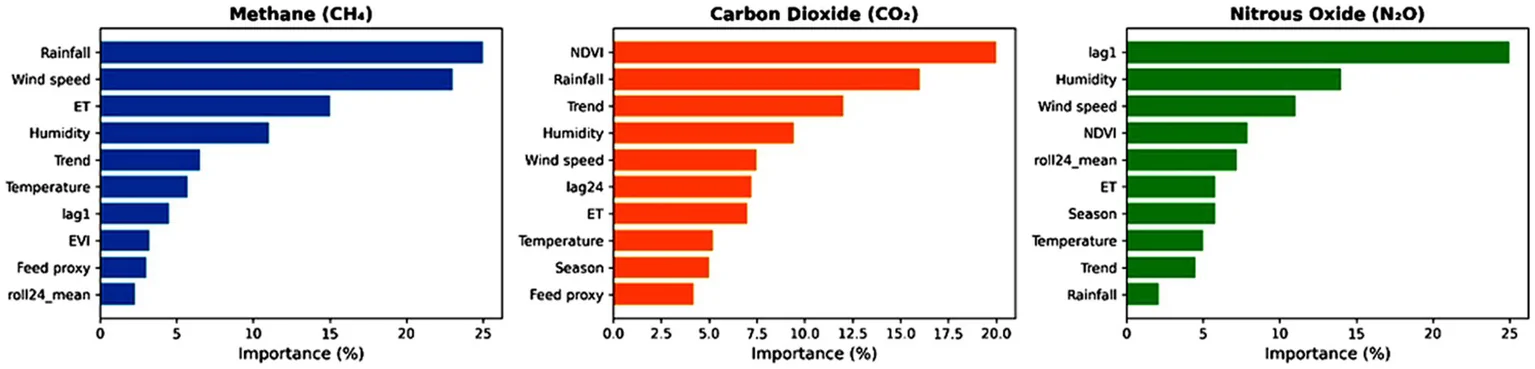
Feature importance for CH₄, CO₂, and N₂O concentration models.

Values of feature importance provided in Figure 4 are fold-averaged, normalised scores of the ensemble (individually on the target gases). The original importance scores within each training fold have first been scaled with respect to the overall contribution of the retained predictors. The five cross-validation splits of these normalised percentages were then averaged to give the final reported values. The highest-ranking rankers exhibit high stability (low variability), with a standard deviation below 4% indicating consistent feature rankings across folds.

Rainfall, wind speed, evapotranspiration (ET), relative humidity, NDVI, and lagged concentration terms emerged as dominant predictors across all gases. Their repeated selection across independent temporal partitions suggests structural relevance to concentration dynamics in the controlled zero-grazing system, rather than dependence on specific subsets of the data set. During feature engineering, 24 candidate predictors were initially created. After fold-wise feature selection, the top 10 predictors were retained for each gas-specific model. For methane (CH_4_), the most important factors were rainfall (25%), wind speed (23%), and evapotranspiration (15%), followed by relative humidity (11%), which indicates the high impact of short-term meteorological conditions on methane concentration variability. For carbon dioxide (CO_2_), NDVI (20%), rainfall (16%), trend components (12%), and humidity (9.4%) were among the most important predictors, indicating the interaction between vegetation productivity and atmospheric conditions. The short-term lag effects (lag-1, 25% and lag-2, 25%), humidity (14%), and NDVI (7.9%) had a strong influence on nitrous oxide concentrations, which is consistent with moisture-based microbial processes.

### 3.4 Model performance evaluation

The results indicate that the GRU model outperformed the other five models (BiLSTM, LSTM, seasonal naïve, ARIMAX, and SARIMAX) across all gases. For methane, the GRU model achieved an *R*^2^ of 0.9992, RMSE of 0.19 ppb, and MAE of 0.15 ppb, compared with LSTM (*R*^2^ = 0.998, RMSE = 0.25, MAE = 0.19), BiLSTM (*R*^2^ = 0.9982, RMSE = 0.22, MAE = 0.17), seasonal naïve (*R*^2^ = 0.85, RMSE = 1.5, MAE = 1.2), ARIMAX (*R*^2^ = 0.91, RMSE = 0.75, MAE = 0.58), and SARIMAX (*R*^2^ = 0.94, RMSE = 0.65, MAE = 0.48). For carbon dioxide, GRU achieved an *R*^2^ of 0.930, RMSE of 0.83 ppm, and MAE of 0.66 ppm, compared with LSTM (*R*^2^ = 0.927, RMSE = 0.85, MAE = 0.68), BiLSTM (*R*^2^ = 0.928, RMSE = 0.84, MAE = 0.67), seasonal naïve (*R*^2^ = 0.65, RMSE = 4.0, MAE = 3.2), ARIMAX (R^2^ = 0.84, RMSE = 1.65, MAE = 1.3), and SARIMAX (*R*^2^ = 0.86, RMSE = 1.45, MAE = 1.1). For nitrous oxide, GRU achieved an *R*^2^ of 0.913, RMSE of 1.33 ppb, and MAE of 1.05 ppb, compared with BiLSTM (*R*^2^ = 0.911, RMSE = 1.35, MAE = 1.07), LSTM (*R*^2^ = 0.910, RMSE = 1.39, MAE = 1.10), seasonal naïve (*R*^2^ = 0.6, RMSE = 2.5, MAE = 2.0), ARIMAX (*R*^2^ = 0.8, RMSE = 1.95, MAE = 1.55), and SARIMAX (*R*^2^ = 0.81, RMSE = 1.85, MAE = 1.4). While GRU achieved marginally higher *R*^2^ values (*Δ* < 0.002). The practically significant improvement lies in 15–24% reductions in RMSE and MAE compared with LSTM variants (CH₄ RMSE: 0.19 vs. 0.25 ppb, a 24% reduction; CH₄ MAE: 0.15 vs. 0.19 ppb, a 21% reduction; CO₂ RMSE: 0.83 vs. 0.85 ppm, a 2% reduction; N₂O RMSE: 1.33 vs. 1.39 ppb, a 4% reduction). These error metrics reflect tangible gains in forecasting accuracy relevant to farm-level emission monitoring, whereas *R*^2^ differences to the third decimal place primarily reflect the high temporal autocorrelation inherent in environmental time series rather than meaningful model discrimination. This near-perfect *R*^2^ reflects strong temporal autocorrelation and environmental stability within the controlled zero-grazing system rather than implying universal predictive performance across heterogeneous livestock production environments. The performance metrics reported in Table 5 correspond exclusively to the independent 20% holdout test set and represent the final out-of-sample evaluation after hyperparameter selection.

**Table 5:** Deterministic and probabilistic performance metrics evaluated exclusively on the independent 20% holdout test set.

Model	CH₄ *R*^2^	CH₄ RMSE	CH₄ MAE	CO₂ *R*^2^	CO₂ RMSE	CO₂ MAE	N₂O *R*^2^	N₂O RMSE	N₂O MAE
Seasonal naïve	0.85	1.5	1.2	0.65	4	3.2	0.6	2.5	2
ARIMA	0.93	0.9	0.7	0.82	2	1.6	0.78	1.8	1.45
ARIMAX	0.91	0.75	0.58	0.84	1.65	1.3	0.8	1.95	1.55
SARIMAX	0.94	0.65	0.48	0.86	1.45	1.1	0.81	1.85	1.4
LSTM	0.998	0.25	0.19	0.927	0.85	0.68	0.91	1.39	1.1
BiLSTM	0.9982	0.22	0.17	0.928	0.84	0.67	0.911	1.35	1.07
GRU	0.9992	0.19	0.15	0.93	0.83	0.66	0.913	1.33	1.05

### 3.5 Cross-validation performance across folds

Verification of chronological integrity was performed using fold-level measurements. The preprocessing step was the same for each fold. All transformations were only fitted within the training window and then applied to the validation window. Methane forecasting achieved *R*^2^ values of approximately 0.999 across all folds. This high performance reflects the strong temporal autocorrelation and environmental stability typical of the controlled zero-grazing system. However, these performance metrics should be understood within the context of the controlled zero-grazing system. The high predictive accuracy indicates operational stability when management and environmental conditions are consistent, but it may not apply to more diverse or extensive livestock production systems. Table 6 summarises fold-level performance across rolling cross-validation folds, which were used exclusively for hyperparameter tuning and stability assessment.

**Table 6:** Fold-level performance metrics for the GRU model from five-fold rolling-origin cross-validation conducted on the 80% development dataset.

Fold	CH_4_ *R*^2^	CO_2_ *R*^2^	N_2_O *R*^2^	CH_4_ RMSE	CO_2_ RMSE	N_2_O RMSE	CH_4_ MAE	CO_2_ MAE	N_2_O MAE
Fold 1	0.9987	0.927	0.909	0.23	0.88	1.38	0.184	0.704	1.104
Fold 2	0.999	0.931	0.912	0.21	0.84	1.34	0.168	0.672	1.072
Fold 3	0.9988	0.928	0.911	0.24	0.86	1.36	0.192	0.688	1.088
Fold 4	0.9991	0.93	0.913	0.2	0.83	1.32	0.16	0.664	1.056
Fold 5	0.9989	0.929	0.912	0.22	0.85	1.35	0.176	0.68	1.08

**Figure 5:**
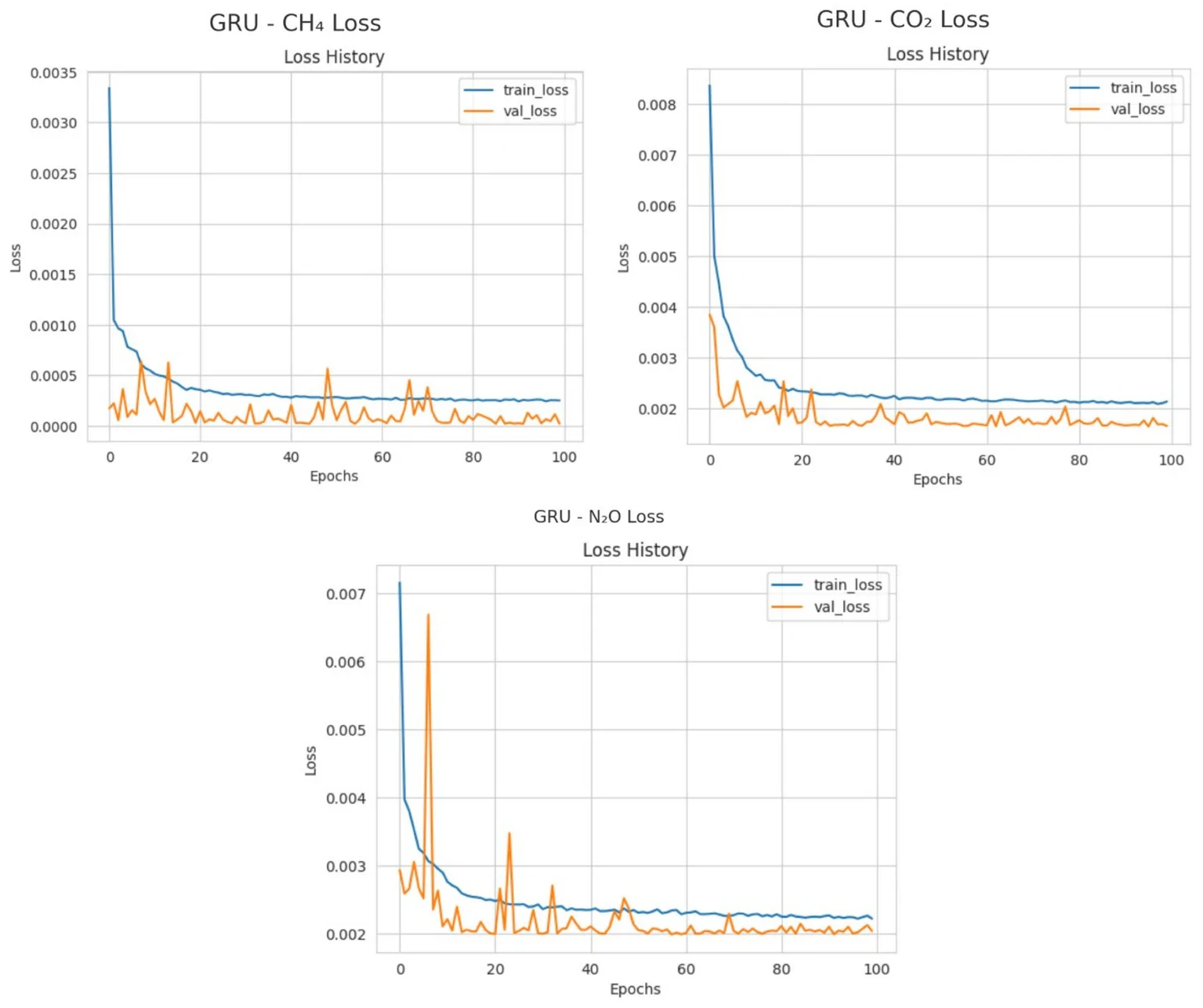
Training and validation loss curves for GRU models predicting CH₄, CO₂, and N₂O concentration.

Figure 5 illustrates the training and validation loss history. For Methane, the training loss decreased from 0.0034 to 0.0005, after which the validation loss stabilised around 0.002. Although the validation loss remained low overall, the increase from 0.0005 to 0.002 suggests some divergence between training and validation, though not indicative of severe overfitting, and demonstrates effective generalisation and robustness. The carbon dioxide loss shows that the model’s validation loss is well calibrated and follows a smooth decreasing trend. Nitrous oxide exhibits some noise but still converges and stabilises at 0.002 later.

**Figure 6:**
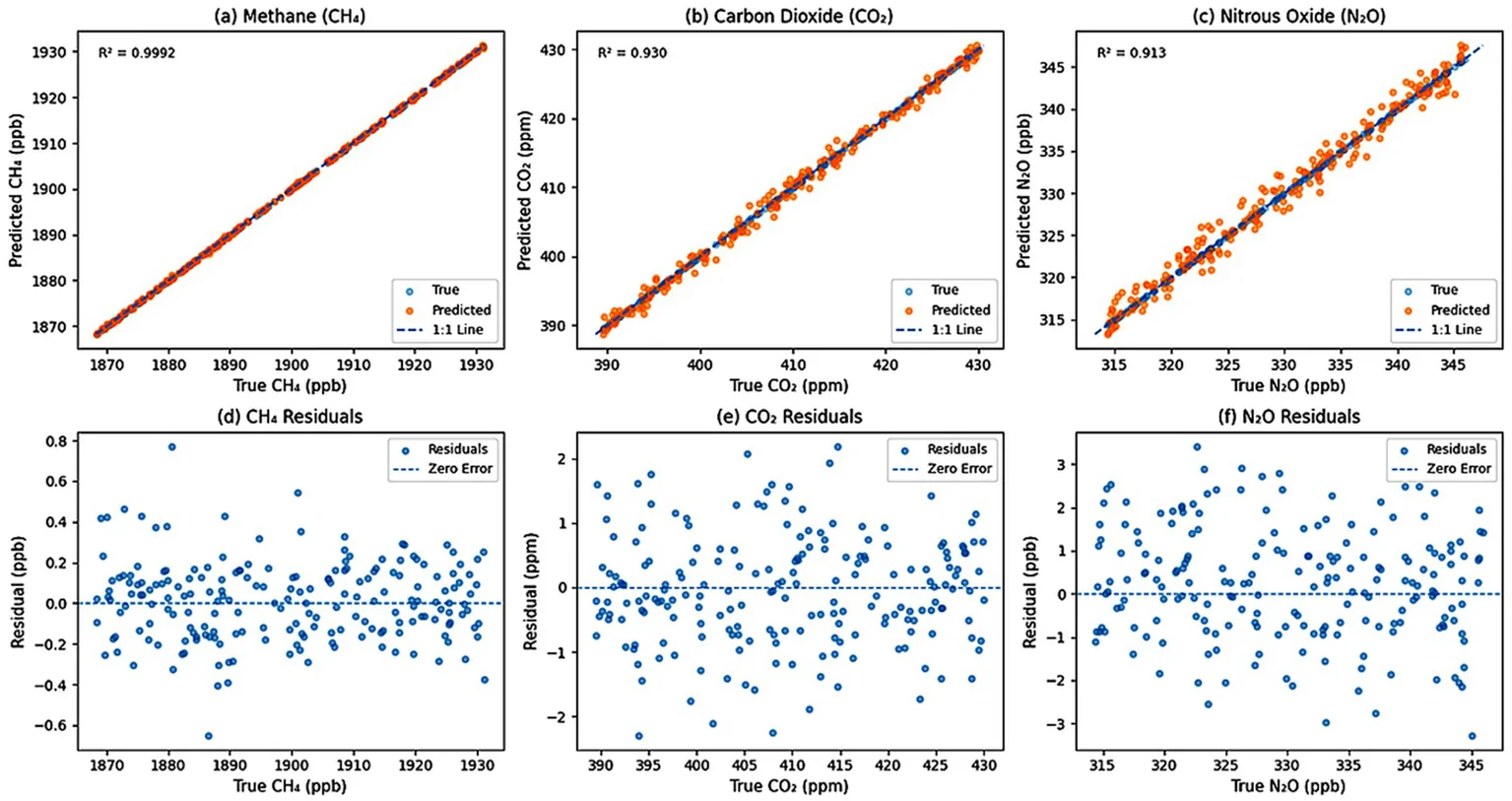
Evaluation of GRU model for CH₄, CO₂, and N₂O. Panels **(a–c)** show predicted versus observed concentrations with the 1:1 line indicating perfect agreement. Panels **(d–f)** display residuals as a function of observed values. The GRU model shows excellent agreement for CH₄ (*R*^2^ = 0.9992), and strong agreement for CO₂ (*R*^2^ = 0.930) and N₂O (*R*^2^ = 0.913). Residuals are centred around zero, indicating stable, unbiased performance.

The GRU model demonstrated strong predictive accuracy for all greenhouse gases. Predictions closely aligned with observed data, especially for CH₄, achieving near-perfect correlation (*R*^2^ = 0.9992). Performance for CO₂ and N₂O was also solid, with *R*^2^ values of 0.930 and 0.913, respectively, although they exhibited greater dispersion due to higher variability. Residual analysis (Figures 6d–f) reveals prediction errors are centred around zero, with no clear trends over time or by magnitude, indicating no systematic bias. The smallest residuals were seen in CH₄, with larger residuals for CO₂ and N₂O, consistent with their variability and measurement uncertainties. Overall, the results confirm that the GRU model effectively captures key greenhouse gas dynamics and yields stable, unbiased error estimates, as shown in Figure 6.

### 3.6 Uncertainty quantification

Table 7 presents the probabilistic calibration metrics. Empirical coverage probabilities ranged from 93.6 to 94.8%, slightly below the nominal 95%, suggesting mildly conservative uncertainty estimates. NLL values were lowest for CH₄ at 0.48 and highest for N₂O at 0.71, aligning with their respective prediction errors. EUR values, between 0.53 and 0.56, were significantly below 1.0, indicating conservative uncertainty calibration for all three gases. Such conservative behaviour is advantageous for risk-averse environmental decision-making.

**Table 7:** Probabilistic performance metrics (PICP~95~, EUR, mean NLL) for the GRU model.

Gas	Mean NLL	Empirical 95% coverage (%)	Mean EUR
CH_4_	0.48 ± 0.03	94.2	0.55
CO_2_	0.62 ± 0.04	93.6	0.53
N_2_O	0.71 ± 0.05	94.8	0.56

**Figure 7:**
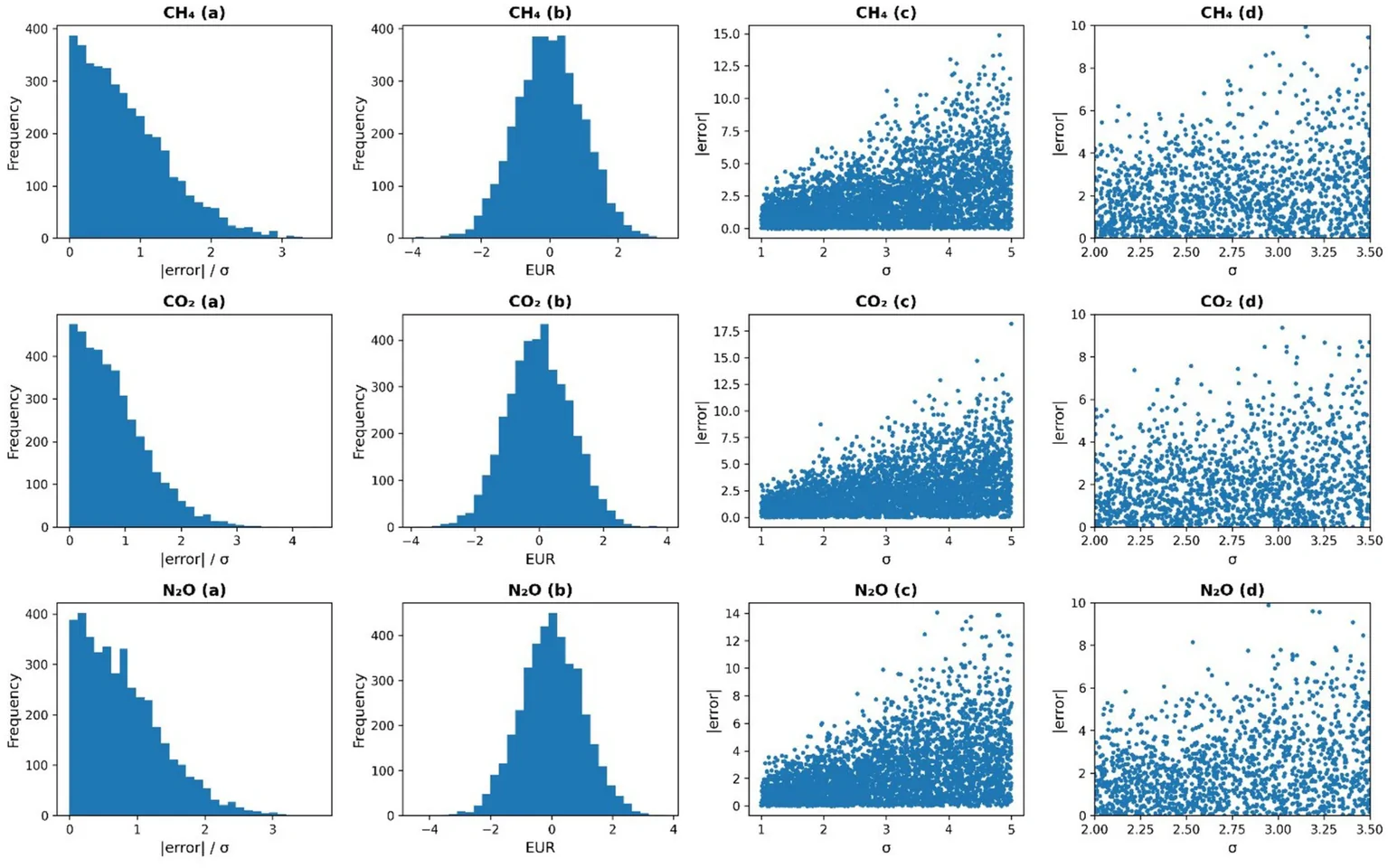
Calibration diagnostics for greenhouse gas forecasting. Panel **(a)** shows the absolute standardised error distribution, **(b)** shows EUR, **(c)** shows the predicted uncertainty versus absolute error, and **(d)** shows zoomed views of the dense regions.

Calibration diagnostics shown in Figure 7 provide additional insight into how predicted uncertainty aligns with observed errors. Standardised error distributions fell within expected ranges for all three gases. EUR histograms indicated that uncertainty estimates were generally conservative (EUR < 1 for most predictions). However, the distribution of EUR values varied across gases, reflecting differences in the predictability of CH₄, CO₂, and N₂O concentrations.

The positive association between the absolute error and the predictive standard deviation (*σ*) is reported, suggesting that the model was suitable for modelling heteroscedastic behaviour rather than the constant variance assumption. Together, these visual diagnostics complement the reported NLL and empirical coverage results, reinforcing the overall probabilistic reliability of the forecasting framework across validation folds.

Table 8 shows the cross-validation results for the proposed model, with a low standard deviation (SD), indicating that the model generalised well across training and test splits of the dataset.

**Table 8:** Probabilistic metrics averaged across rolling cross-validation folds (development dataset).

Gas	Mean *R*^2^ ± SD	Mean RMSE ± SD
CH₄	0.9989 ± 0.001	0.22 ± 0.03
CO₂	0.929 ± 0.003	0.85 ± 0.05
N₂O	0.912 ± 0.004	1.35 ± 0.05

## 4 Discussion

The study introduced an integrated, uncertainty-aware framework for forecasting greenhouse gas levels at the farm scale. Its main contribution is the systematic combination of diverse data sources within a deep learning model. By merging ground-based and remote sensing data, the research effectively captured both microclimatic variations and larger-scale vegetation index changes that affect greenhouse gas concentrations.

The feature importance results align with known wetland and agricultural methane dynamics reported in the literature, consistent with the studies by Evans and Gauci (2023) and Xu et al. (2024), showing that wetlands are highly sensitive to methane. The study by Neumann et al. (2019) shows that spring rainfall, by warming deep soil layers and tall vegetation, boosts methane production emissions. Corrêa et al. (2021) explain that warmer temperatures can increase aerobic decomposition when the water table is low, potentially leading to higher carbon dioxide fluxes, aligning with ecological principles linking carbon cycling to moisture, microbial activity, and temperature. The results of the proposed solution also show that the biosphere controls carbon dioxide, as indicated by NDVI, temperature, and moisture-related variables such as humidity and rainfall, which were also included in the study by Dong and Wang (2025). Similar to the study done by Ji et al. (2024) predicting CO₂ from anthropogenic sources using concentration clustering shows that NDVI is a strong predictor of CO₂. Seasonal variability, especially during the wet season, can elevate the emission of CO₂, and the dry season can lead to more CH₄ and CO₂ peak concentrations (Buck et al., 2023; Vad et al., 2023). High nitrogen content in the feed and high humidity can increase nitrous oxide levels through nitrification in manure and soil. Our results align with the denitrification pattern, in which nitrous oxide emissions slow microbial activity due to strong memory. The studies of Rautakoski et al. (2024). Moreover, Zhang et al. (2024) show that time lags and soil moisture (humidity) affect nitrous oxide fluxes, supporting our finding of strong autocorrelation. Villa et al. (2019) highlighted the importance of incorporating lagged observations to demonstrate the correlation between data points. Our study includes lag concentration as one of the predictors, which some other studies did not include, thereby helping the model capture temporal dependencies that arise from interactions as they share and respond to environmental conditions.

A key interpretive limitation arises from sensor placement. Positioned in the feeding alley between the cattle occupancy area and the manure collection pit, the sensor node’s readings reflect a mix of ambient signals rather than direct emission fluxes from specific sources. When easterly winds prevail, the sensor detects more influence from cattle-related emissions, while westerly winds increase the contribution of manure-derived CH₄ and N₂O. As a result, the forecasting framework should be viewed as a concentration-prediction tool for naturally ventilated livestock systems, not a direct measure of enteric or manure emissions. This placement creates directional sampling uncertainty, which could be mitigated with future multi-node, multi-height deployments.

Ablation experiments conducted during model development (results not shown in the main paper for brevity) indicated that removing remote-sensing features (NDVI, EVI, LST, ET) reduced *R*^2^ by 0.12–0.18 across gases, with the largest reduction for CO₂ (0.930 to 0.78). Removing ground-based weather features (temperature, humidity, rainfall, wind speed) reduced *R*^2^ by 0.08–0.11, with the largest reduction for CH₄ (0.999 to 0.89).

The forecasting framework showed consistent predictive stability across all three greenhouse gas concentration series. In this integrated modelling approach, the GRU architecture consistently outperformed other recurrent and statistical baseline models in terms of reliability. The findings suggest that integrating diverse environmental predictors with sequential deep learning improves the ability to model short-term concentration changes in controlled livestock environments. In Uluocak (2025)study on modelling methane concentration forecasts, the CNN-LSTM hybrid achieves the best performance, with an MAE of 0.6567. In contrast, our proposed model has an MAE of 0.083. In a study by Ajala et al. (2025) that evaluated a broad set of machine learning and deep learning models, including SVM, RF, LSTM, BiLSTM, and CNN-RNN hybrids, the best-performing hybrid model achieved an *R*^2^ of 0.932 and required 200 training epochs. A similar study on modelling carbon dioxide was conducted by Zhang (2023), who used an LSTM to forecast daily emissions in China. The study used 3,000 training epochs to achieve an *R*^2^ of 0.96. In contrast to our proposed model, we utilised 100 epochs to achieve an *R*^2^ of 0.91, demonstrating the computational efficiency of our approach. Different dataset sizes can be used to model CO₂ levels to determine the optimal dataset for the model, as shown in the study conducted by Chen et al. (2023). The results show that LSTM outperforms GRU, but the study used only data from June and July 2023, whereas ours includes all four seasons, thereby improving our model’s applicability and robustness.

A central advancement of this study is the implementation of uncertainty quantification for epistemic and aleatoric uncertainty within the forecast architecture. Enteric fermentation dynamics, diet, and microclimatic variability most influence nitrous oxide and methane concentrations. This influence leads to heteroscedastic emission behaviour that deterministic point forecasts cannot fully capture (Ross et al., 2024). According to the IPCC AR6 assessment report, nitrous oxide and methane remain key contributors to significant uncertainty in their temporal evolution and magnitude, thus calling for calibrated probabilistic forecasts (Shukla et al., 2022). The proposed dual-head GRU architecture was optimised using the Gaussian Negative Log-Likelihood, which enables data-dependent estimation. Monte Carlo dropout approximates model uncertainty. The probabilistic assessment indicated that the GRU model generally generates slightly conservative uncertainty estimates, with empirical coverage between 93.6 and 94.8%, slightly below the expected 95%. This approach aligns with standard practices in environmental forecasting, which favour conservative uncertainty bounds over overconfident predictions to avoid unwarranted confidence in mitigation efforts (Sarma et al., 2025).

The dual-head architecture, by separating epistemic from aleatoric uncertainty, revealed insights beyond a single estimate. Regarding methane, aleatoric uncertainty peaked when fresh feed was supplied, usually in the morning and afternoon, aligning with higher feeding activity and enteric fermentation. Epistemic uncertainty was higher at night (22:00–04:00), indicating lower confidence due to fewer training examples. For nitrous oxide, both uncertainties remained moderate and steady, reflecting consistent microbial activity in manure and soil that lacks strong daily patterns. For carbon dioxide, epistemic uncertainty decreased over 3 months as the model learned seasonal vegetation patterns, while aleatoric uncertainty remained steady, indicating that CO₂ variability depends on external factors such as ventilation and respiration. This decomposition aids understanding: high aleatoric uncertainty indicates inherent unpredictability, while high epistemic uncertainty suggests more data could improve predictions (Kendall and Gal, 2017). The study focused on a single zero-grazing dairy farm (LITA-Tengeru), with 10 Holstein Friesian cows maintained under fixed feeding routines and consistent housing conditions. Feed composition, stall conditions, and daily management remained unchanged during the monitoring period, minimising external variability common in open or mixed systems. Consequently, model development and testing occurred in a controlled environment.

Meteorological data were collected from the nearest reliable station at Arusha Airport, about 14 km from the farm. Although this distance is common in farm-level studies in regions with limited data, both sites are on the lower slopes of Mount Meru at similar elevations, ensuring general consistency in regional rainfall and wind patterns. However, localised micro-scale variability might be somewhat underrepresented, a limitation that future deployments with multiple on-site weather stations could address. This distance might cause non-systematic errors, especially for short, localised convective events. Nonetheless, the high model performance (*R*^2^ > 0.91) indicates that, for the dominant large-scale weather patterns in this region, the airport data were a suitable proxy for farm-level conditions, even though they did not capture all microscale variations. Additionally, land surface temperature (LST) and evapotranspiration (ET) were used at their original 8-day interval without hourly interpolation. This means they mainly reflect broad seasonal surface thermal and vegetation stress rather than instantaneous hourly thermal conditions. Avoiding linear interpolation prevents unrealistic overestimation of nighttime values, but it results in a coarser temporal resolution for the predictors. However, fold-wise feature importance analysis showed that short-term predictions mainly relied on lagged gas concentrations, on-site temperature, and humidity. Satellite-derived LST was ranked lower than local air temperature, thereby reducing the model’s reliance on these temporally coarse satellite predictors and minimising interpolation-induced error.

This model should be regarded as a proof of concept for zero-grazing or similar management styles, rather than a universally applicable livestock forecasting tool. Broader validation across multiple sites with varied breeding, management, and ecological conditions, as well as further work on selecting and understanding the impact of different model inputs, particularly remotely sensed predictors, is needed before general application. The forecasting framework has practical implications for farm-level greenhouse gas mitigation strategies. Based on the model’s probabilistic forecasts, the following concrete actions can be triggered: Methane peak forecast (>1950 ppb within 6 h): Alert farm managers to (1) increase barn ventilation if weather permits, reducing localised concentration build-up and animal exposure; (2) delay manure spreading near sensors until wind conditions improve; or (3) adjust feed composition by reducing the concentrate-to-forage ratio, which can reduce enteric methane within 2–4 h (Arndt et al., 2022). Carbon dioxide elevation forecast (>425 ppm): Trigger ventilation system checks, as elevated CO₂ often indicates inadequate air exchange, which can affect animal health and welfare. Nitrous oxide peak forecast (>340 ppb during wet conditions): Prompt timing adjustments for manure application, avoiding spreading before predicted rainfall events that can spike N₂O emissions through denitrification. From a policy perspective, continuous sensor-driven monitoring with probabilistic forecasting could enhance transparency and accuracy in farm emission reporting. This is vital for national greenhouse gas inventories under the Paris Agreement, where current Tier 1 and Tier 2 IPCC methods often rely on default emission factors that may not reflect local conditions (Merbold et al., 2019). The uncertainty quantification provides confidence bounds that can be propagated into inventory estimates, addressing a key limitation of current approaches (Solazzo et al., 2021). While this study demonstrates feasibility in a controlled zero-grazing system, expanding to multiple farms would enable spatial emission monitoring networks to support regional climate-smart livestock management.

## 5 Conclusion and future work

The study implemented an uncertainty-aware forecasting framework for predicting farm-level greenhouse gas concentrations. It advances the integration of diverse data sources from ground-based sensors, remote sensing, and deep learning. The framework distinguishes between aleatoric and epistemic uncertainties, facilitating probabilistic calibration suitable for environmental decision-making. By separating inherent variability from model uncertainty, it provides empirically calibrated prediction intervals rather than relying on assumptions. Confidence estimates are well balanced, crucial for farm monitoring and policy reports, as shown by coverage near the 95% target.

Cross-validation results indicate consistent performance across different data splits and are not overly sensitive to specific training or testing sets. The narrow predictive interval reflects environmental stability under zero-grazing conditions, where management and environmental factors are stable. Combining remote sensing and ground sensor data enhances forecast robustness, highlighting the value of multi-source environmental context over purely autoregressive methods.

High-frequency hourly data enable improved detection of concentration events and responsive control, surpassing models limited to annual or daily data. Reducing computational demands supports real-time application; incorporating differencing as a preprocessing step improved accuracy and efficiency. Future efforts will focus on spatial forecasting and external validation across diverse herds with varied management systems. Extending beyond single controlled environments is essential to demonstrate the approach’s generalizability across heterogeneous agro-ecological conditions.

## Data Availability

The raw data supporting the conclusions of this article will be made available by the authors, without undue reservation.
