# Correction: Gated recurrent unit model for forecasting greenhouse gas concentrations with uncertainty quantification

**DOI:** 10.3389/frai.2026.1954403

**Published:** 2026-08-20

**Authors:** Erica Hargety Kimei, Devotha Godfrey Nyambo, Neema Mduma, Shubi Felix Kaijage

**Affiliations:** 1 School of Computation and Communication Science and Engineering, The Nelson Mandela African, Institution of Science and Technology, Arusha, Tanzania; 2 Informatics and Technical Education, National Institute of Transport, Dar-Es-Salam, Tanzania

**Keywords:** forecasting, GHG, greenhouse gas concentration, GRU, IoT, remote sensing, uncertainty quantification

The funder UK International Development and the International Development Research Centre, Ottawa, Canada was erroneously omitted.

The correct **Funding** statement reads:

The author(s) declared that financial support was received for this work and/or its publication. This work was carried out with financial support from UK International Development and the International Development Research Centre, Ottawa, Canada as part of the AI for Development: Responsible AI, Empowering People Program (AI4D).

The original version of this article has been updated.

